# Lower Baseline Germinal Center Activity and Preserved Th1 Immunity Are Associated With Hepatitis B Vaccine Response in Treated HIV Infection

**DOI:** 10.20411/pai.v2i1.175

**Published:** 2017-03-14

**Authors:** Robert M. Paris, Lucimar G. Milagres, Eirini Moysi, Jason F. Okulicz, Brian K. Agan, Anu Ganesan, Constantinos Petrovas, Richard A. Koup

**Affiliations:** 1 US Military Malaria Research Program, Walter Reed Army Institute of Research, Silver Spring, Maryland; 2 Immunology Laboratory, Vaccine Research Center, NIAID, NIH, Bethesda, Maryland; 3 Department of Microbiology and Immunology, Universidade do Estado do Rio de Janeiro, Rio de Janeiro, Brazil; 4 Tissue Analysis Core, Immunology Laboratory, Vaccine Research Center, NIAID, NIH, Bethesda, Maryland; 5 Infectious Diseases Service and HIV Medical Evaluation Unit, San Antonio Military Medical Center, San Antonio, Texas; 6 Infectious Diseases Clinical Research Program, Department of Preventive Medicine and Biostatistics, Uniformed Services University of the Health Sciences, Bethesda, Maryland; 7 Henry M. Jackson Foundation for the Advancement of Military Medicine, Bethesda, Maryland

**Keywords:** Human immunodeficiency virus, AIDS, hepatitis B vaccine, T cell, antibodies

## Abstract

**Background:**

Why HIV-infected individuals have poor responses to standard dose and schedule hepatitis B virus immunization is not well understood.

**Methods:**

We compared the serologic and cellular immune profiles of treated HIV-infected individuals with similar durations of infection and preserved CD4 counts (> 350 cells/microliter) by hepatitis B vaccine (HBV) response before and after vaccination.

**Results:**

Similar levels of immune activation and plasma cytokine profile were found between non-responders and responders. The baseline plasma levels of CXCL-13, a surrogate of germinal center reactivity, were significantly lower in HBV responders compared to HBV non-responders and were a predictor of both vaccine response and titer. Furthermore, response to HBV vaccination was associated with a significantly higher frequency of circulating IgG^high^ memory B cells post vaccination and preserved Th1 antigen-specific T-cell responses.

**Conclusions:**

Taken together, our data suggest that preserved Th1 responses are associated with hepatitis B vaccine response in treated HIV infection.

## INTRODUCTION

Approximately 5-10% of healthy adults have an inadequate response to three doses of hepatitis B vaccine [1], which is associated with human leukocyte antigen (HLA) class II polymorphism [2, 3]. Hepatitis B vaccine (HBV) non-response (defined as hepatitis B surface antibody < 10 mIU/ mL after completion of the three-immunization series) is even more common in HIV infection [4, 5], and immune reconstitution with combination antiretroviral therapy (cART) appears to have less impact on this response [4, 6]. This is in contrast to influenza vaccination where vaccine response and protection are associated with CD4 count and cART [7–10]. Even with a four-vaccination, double-dose immunization regimen, a significant number of HIV-infected individuals failed to demonstrate an adequate immune response [11]. Importantly, the timing of vaccination relative to HIV infection (before vs after) appears to impact protection from hepatitis B infection: Landrum *et al*. documented significantly more hepatitis B breakthrough infections among HIV-infected individuals who were vaccinated after HIV infection and despite an HBV titer greater than 10 mIU/ml [4]. These findings suggest that the immune response to HBV among HIV-infected individuals may be qualitatively different than the immune responses to influenza or other subunit vaccines. The inability to mount or maintain memory B-cell responses is a well-recognized phenomenon in chronic HIV infection, and a number of studies have demonstrated the loss of serological memory to immunization [12] and CD27^high^ memory B cells [13, 14], which most likely begins at the time of infection and continues throughout the course of the disease (reviewed in [15]). The concurrent loss of both T-dependent and T-independent antigen-specific responses suggests a direct effect of HIV on B-cell populations and both direct and indirect effects on T-B cell interactions [14]. Potential mechanisms for B-cell dysfunction include direct viral effects on B-cells as well as polyclonal activation, resulting in B-cell exhaustion and altered homing [16], and impaired class switch recombination and affinity maturation [17]. In contrast, T-cell phenotype and function in HBV vaccination is not well studied. In the last decade, seminal observations of follicular T-B cell interactions have revealed a unique set of T cells that provides direct B-cell help [18] within secondary lymphoid tissues, referred to as T follicular helper cells (Tfh). These cells are typically characterized by expression of CXCR5 and PD-1 and antigen-specific IL-21 production. Tfh cells appear to develop in association with increased Bcl-6 transcription, which suppresses canonical T helper cell developmental pathways (that is, Th1, Th2, Th17, etc.). Though peripheral Tfh cells are not well defined, we have observed that there are subsets of circulating T-cells with a central memory phenotype that express high levels of CXCR5, SLAM (CD150), and CCR6, and dim or low expression of PD-1. These are associated with the magnitude and breadth of class-switch recombination in T-B cell co-culture experiments, decrease with HIV disease progression, and may be lost early during the course of HIV infection [19, 20].

The cellular immunology of hepatitis vaccine response in treated and/or well-controlled HIV infection is not well studied and may provide further insight into the optimization of antigen dose, adjuvant selection, and dosing interval. Here we examined T- and B-cell phenotype and function and plasma cytokine profiles in a well-defined cohort of HIV-infected hepatitis B vaccine responders and non-responders [4] in order to characterize the nature and quality of the immune response to HIV-infection and HBV, with the goal of identifying some of the basic mechanisms of B-cell and T-cell biology. We hypothesized that preserved cellular immune phenotype profile and functionality (as measured by antigen-specific cytokine production) are associated with HBV response.

## MATERIALS AND METHODS

### Study Subjects

Twenty individuals were identified from the U.S. Military HIV Natural History Study cohort [21, 22] with the following inclusion criteria: (1) past or current use of cART; (2) CD4 count (> 350 cells/μl); (3) no evidence of hepatitis B infection (hepatitis B surface antigen, surface antibody, and core antibody negative); (4) with concurrent frozen peripheral blood mononuclear cells (PBMC) and plasma collected prior to hepatitis B immunization (standard immunization schedule) and within 12 months after the third vaccination. All subjects provided informed consent prior to participation in the prospective cohort study for which collection of stored blood specimens was utilized for performance of assays in this study. Human experimentation guidelines of the United States Department of Health and Human Services were followed in the conduct of clinical research, under a protocol reviewed and approved by the Institutional Review Boards of the National Institutes of Health and Uniformed Services University, both in Bethesda, Maryland.

### Laboratory Assays

#### Polychromatic flow cytometry.

*Phenotypic analysis:* PBMCs were cultured in RPMI 1640 (Invitrogen) supplemented with 10% fetal bovine serum, 2mM L-glutamine, 100U/mL penicillin, and 100 ug /mL streptomycin (Invitrogen). 1-2 x 10^6^ PBMCs were incubated with Aqua viability dye and surface stained with titrated amounts of antibodies to panel (1): CD3; CD4; CD27; CD45RA; CD127; CCR7; PD-1; CCR6; CXCR5; and ICOS followed by intracellular staining for CTLA-4 and Ki-67 or (2) CD3; CD19; CD20; CD27; CD38; IgD; IgG; IgM; CXCR5; and CCR7. *Intracellular cytokine staining:* 3 x 10^6^ PBMCs were rested for two hours, then incubated for six hours in 1mL of medium containing brefeldin A (10ug/mL) in the absence or presence of HIV-1 subtype B Gag-peptide pools (15mers overlapping by 11 residues; National Institutes of Health AIDS Research and Reference Reagent Program), hepatitis B surface antigen peptide pools (hepatitis B virus Genotype A2/strain adw2, 15 mers overlapping by 11 residues, Proimmune, Sarasota, FL, USA), or Cytostim™ 20μl/ml PBMC suspension (positive control for TCR activation and intra-cellular cytokine production) (Miltenyi Biotech, Auburn, CA). After washing, cells were surface stained with Aqua, CD4, CD8, CD27, CD45RO, CD127, PD-1, and CXCR5. Cells were then washed again, permeabilized (Cytofix/Cytoperm kit; BD Biosciences), and stained with antibodies to CD3, CD154, IFN-γ, IL-2, TNF-α, and IL-21. After fixation with 1% paraformaldehyde, events were collected on a modified LSRII flow cytometer (BD Immunocytometry Systems). Electronic compensation was performed with antibody capture beads (BD Biosciences) stained separately with antibodies used in the test samples. Data were analyzed using FlowJo Version 9.6 (TreeStar, Ashland, OR).

#### Antibodies.

Flow cytometry was performed using the following directly conjugated antibodies: (1) BD Biosciences: CD3 H7APC (SK7), CD45RA Cy7PE (L48), CD20 APC-H7 (2H7), CD45RO TRPE (UCHL), CTLA-4 APC (BNI3), IgD PE (IA6-2), IgG APC (G18-145), IgM PE-Cy5 (G20-127), IL-21 Alexa 647 (3A3-N2.1), and Ki-67 Alexa700 (150503); (2) Beckman Coulter: CD127 Cy5PE (R34.34); (3) BioLegend: PD-1 BV711 (EH12.2H7), IFNg BV421 (4S.B3), TNF-α CY7PE (MAb-11), IL-2 Alexa-700 (MQ1-17H12), CCR6 BV785 (G034E3), CD27 Alexa594 (IA4CD27), CD27 BV605 (O323), CCR7-BV605 (G043H7), CD8 BV785 (SK1), ICOS Pac Blue (C398.4A), CD19 Pac Blue (HIB19), CD38 BV785 (HIT2), and CD154 PE (24-31); (4) Invitrogen: CD4 Cy5.5PE (S3.5) and Aqua LIVE/DEAD^®^ amine viability dye; and (5) eBioscience: CXCR5-FITC (MU5UBEE).

#### Measurement of plasma cytokines and antibody titers.

Plasma or serum samples from subjects at the same time points before and after vaccination were measured for soluble CD14 (sCD14), CXCL-13 using standard ELISA assays according to manufacturer's instructions (Quantikine^®^ ELISA, R & D Systems, Minneapolis, MN) and for the following chemokines and cytokines: IL-2, IL-4, IL-6, IL-10, IL-12p70, IL-15, IL-17A, IL-21, TNF-α, and IFN-γ using the Luminex xMap platform multiplex assay (EMD Millipore, Billerica, MA). TNF-α and IL-6 levels were furthered confirmed by standard ELISA kits (R&D). Quantitative assessment of hepatitis B surface antibody titer was performed using the MONOLISA™ assay (BioRad, Hercules, CA) according manufacturer instructions.

#### Statistical analysis.

Experimental variables were analyzed using nonparametric statistical tests of inference: Mann-Whitney U test; the Wilcoxon matched-pairs signed rank test; or the Kruskal-Wallis test with Dunn's multiple comparison post-test as appropriate. Correlation analysis was performed using the nonparametric Spearman test. Statistical analyses were performed with GraphPad Prism (GraphPad Software, version 5.0) or Stata Statistical Software, Release 11 (StataCorp, College Station, TX).

## RESULTS

### Baseline plasma levels of CXCL-13 are a correlate of vaccine-elicited antibody titers

**Figure 1.**
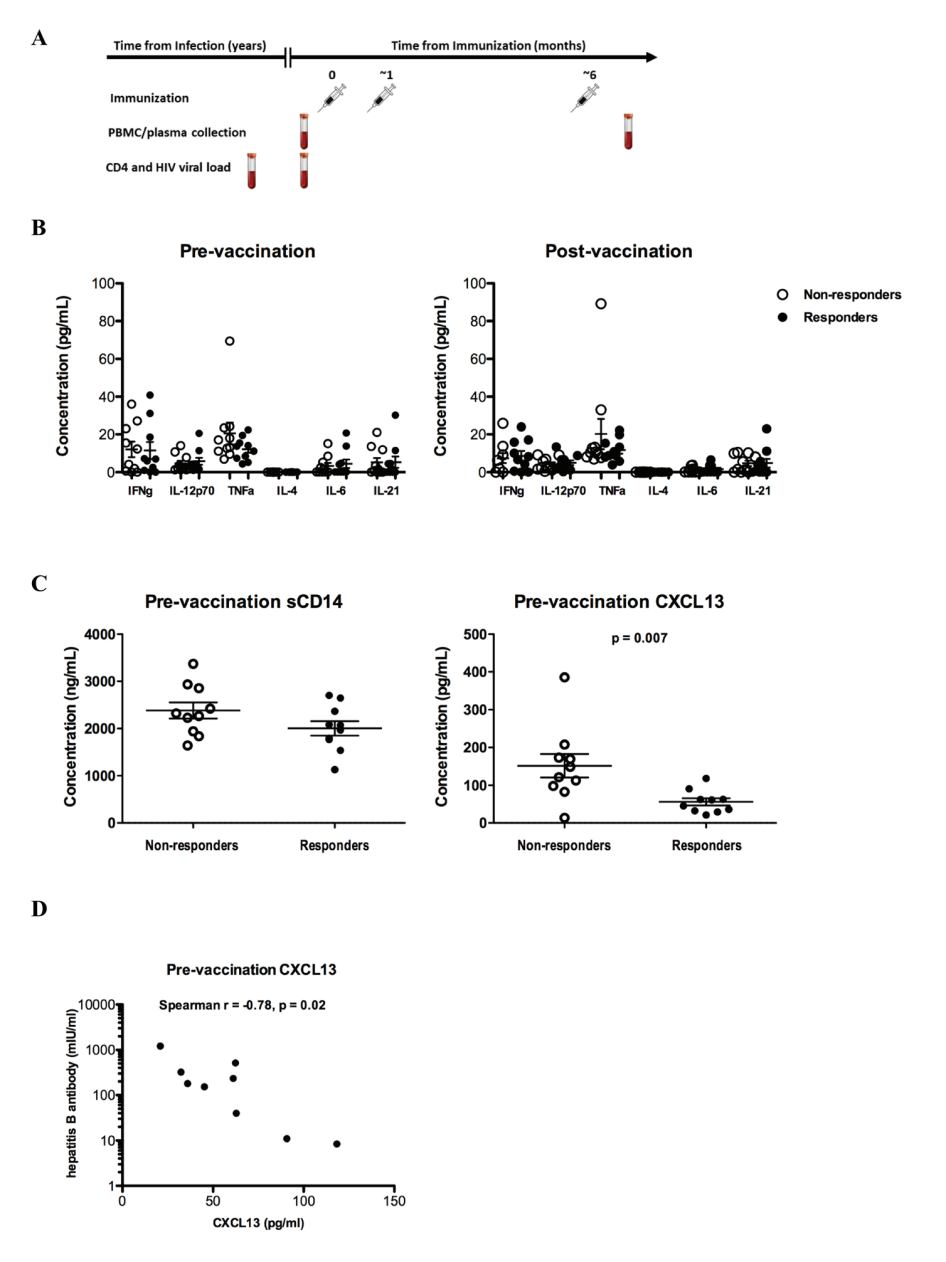
Baseline plasma CXCL-13 levels predict the response to HBV vaccination (A) HIV-infected subjects with and without detectable antibody responses were assessed before and after hepatitis B vacci-nation, (B) Pooled data showing the levels of several cytokines measured in plasma samples from non-responders (n = 10) and responders (n = 10), (C) soluble CD14 levels in non-responders and responders are shown in the *left panel*, while circulating levels of the chemokine CXCL-13 pre-vaccination are shown in the *right panel*, (D) hepatitis B surface antibody (HbSAb) titer among responders post-vaccination. Figures are scatterplots with mean and SEM with only statistically significant *P*-values shown as determined by the Mann-Whitney U test (C), and Spearman rank correlation (D).

**Supplementary Figure 1.**
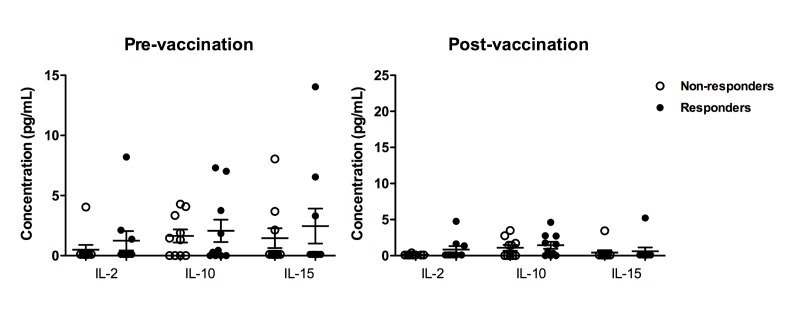
Scatterplot comparison of additional cytokines (IL-2, IL-10, and IL-15) for HBV responders compared to non-responders.

We utilized a natural history cohort of HIV-infected participants in a longitudinal study that has prospectively enrolled ~5,000 subjects since 1986 to identify individuals on treatment, with appropriately timed blood draws corresponding to hepatitis B vaccination that included both plasma and PBMC specimens [21, 23, 24]. HIV-infected subjects included in this analysis (Figure 1A and Supplementary Table 1), classified as hepatitis B vaccine (HBV) responders (n = 10) and non-responders (n = 10), were not statistically different in terms of, age, median CD4 count (median 600.5 vs 504), HIV viral RNA level (median log10 copies/mL, 1.69 vs 1.73), or duration of HIV infection (mean 40.0 months vs 43.1), but were mostly men (all non-responders and 7 responders, *P* = 0.2, Fisher's exact test). A similar profile of plasma cytokines was found between the non-responders and responders both pre- and post-vaccination (Figure 1B and Supplementary Figure 1). Although not significantly, increased levels of the pro-inflammatory cytokine tumor necrosis factor-alpha (TNF-α) was found in non-responders (Figure 1B). There was no difference in general immune activation as measured by soluble CD14 [25] before (Figure 1C, *left panel*) or after vaccination (data not shown) between HBV responders and non-responders. It has been recently shown that plasma levels of CXCL-13 represent a biomarker for the germinal center immunoreactivity [26]. Baseline (pre-vaccination) plasma levels of CXCL-13 were significantly higher (*P* = 0.007, Mann-Whitney test) in non-responders compared to responders (Figure 1C, *right panel*). Moreover, a significant negative correlation was found between baseline plasma levels of CXCL-13 and vaccine-induced antibody titer (Figure 1D). A single outlier whose titer was measured more than one year after vaccination was excluded from this analysis. Although the sample size of this study precludes identification of small differences in plasma cytokines, our data indicate that a lower germinal center reactivity pre-vaccination positively impacts HBV vaccine response in treated HIV infection.

### Vaccine response is associated with increased IgG^high^ memory B cells post vaccination

**Figure 2.**
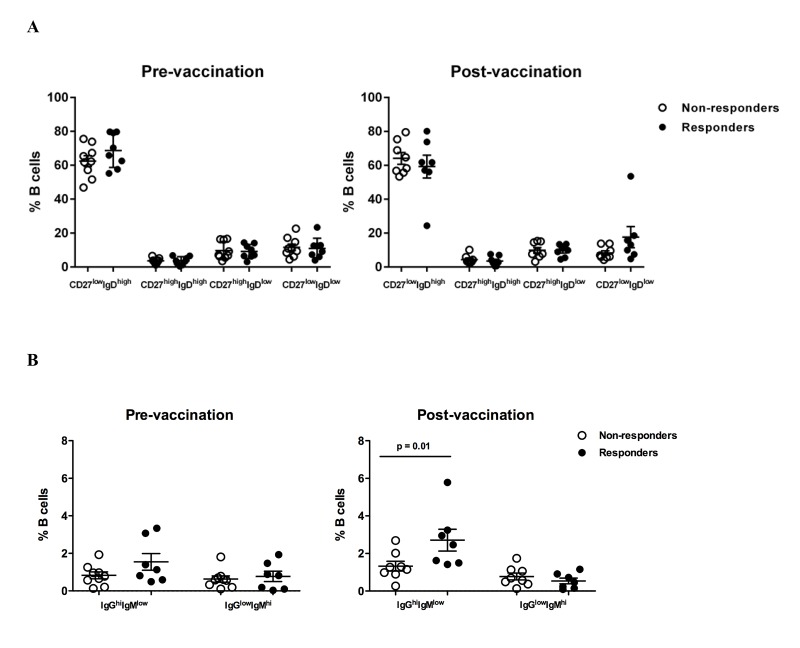
HBV response is associated with increased frequency of IgG^high^ memory B cells post vaccination. (A) Pooled data showing the relative frequencies of B-cell populations, judged by the expression of CD27 and IgD, in non-responders and responders pre- and post-vaccination, (B) the relative frequencies of IgG-^high^IgM^low^ switched memory (CD27^high^IgD^low^) B cells pre- and post-vaccination. Data presented in Figures A and B are scatterplots with mean and SEM with *P*-values calculated using Mann-Whitney U test.

**Supplementary Figure 2.**
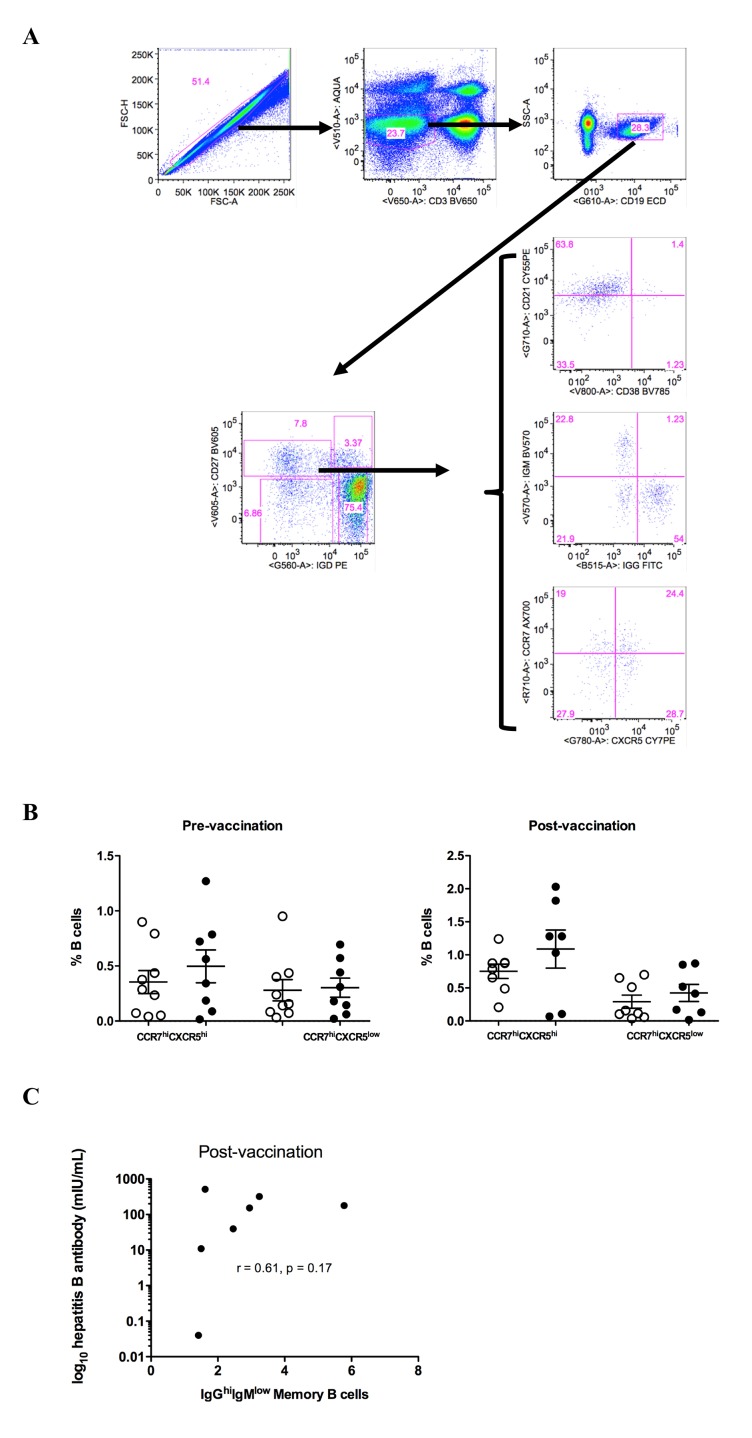
(A) Gating scheme for phenotypic analysis of B cell subsets, (B) Pooled data showing the relative frequency of circulating memory B cells with respect to expression of CCR7 and CXCR5 chemokine receptors and (C) Scatterplot of HBV antibody concentration and the frequency of IgG^high^IgM^low^ switched memory B cells (only 7 HBV responding donors had a measurable response). *P*-values calculated with Mann-Whitney test and (B) and Spearman correlation test (C).

Next, the relative frequencies of circulating B cell populations were investigated pre- and post-vaccination. No differences in terms of B cell differentiation, judged by CD27 and IgD expression, were found between non-responders or responders both pre- and post-vaccination (Figure 2A and Supplementary Figure 2A). No differences were found between responders and non-responders when B-cell populations defined by the expression of chemokine receptors (CCR7, CXCR5) important for B-cell trafficking in LNs [27, 28] (Supplementary Figure 2A and B) or CD21 (or CD27) and CD38 were analyzed (data not shown). Although no difference was found pre-vaccination, a significantly higher (*P* = 0.01) frequency of circulating IgG^high^IgM^low^C D27^high^IgD^low^ “switched” memory B cells was found in responders compared to non-responders post-vaccination (Figure 2B, gating shown in Supplementary Figure 2A). Although the cohort size was underpowered to assess the potential correlation, a positive association between IgG-_high_IgM^low^CD27^high^IgD^low^ B cells and vaccine titer was found (Supplementary Figure 2C). Our data indicate that vaccine responses are associated with induction of particular memory B cells capable of producing antibodies.

### Circulating Tfh cells correlate with plasma levels of CXCL-13 in HBV responders

**Figure 3.**
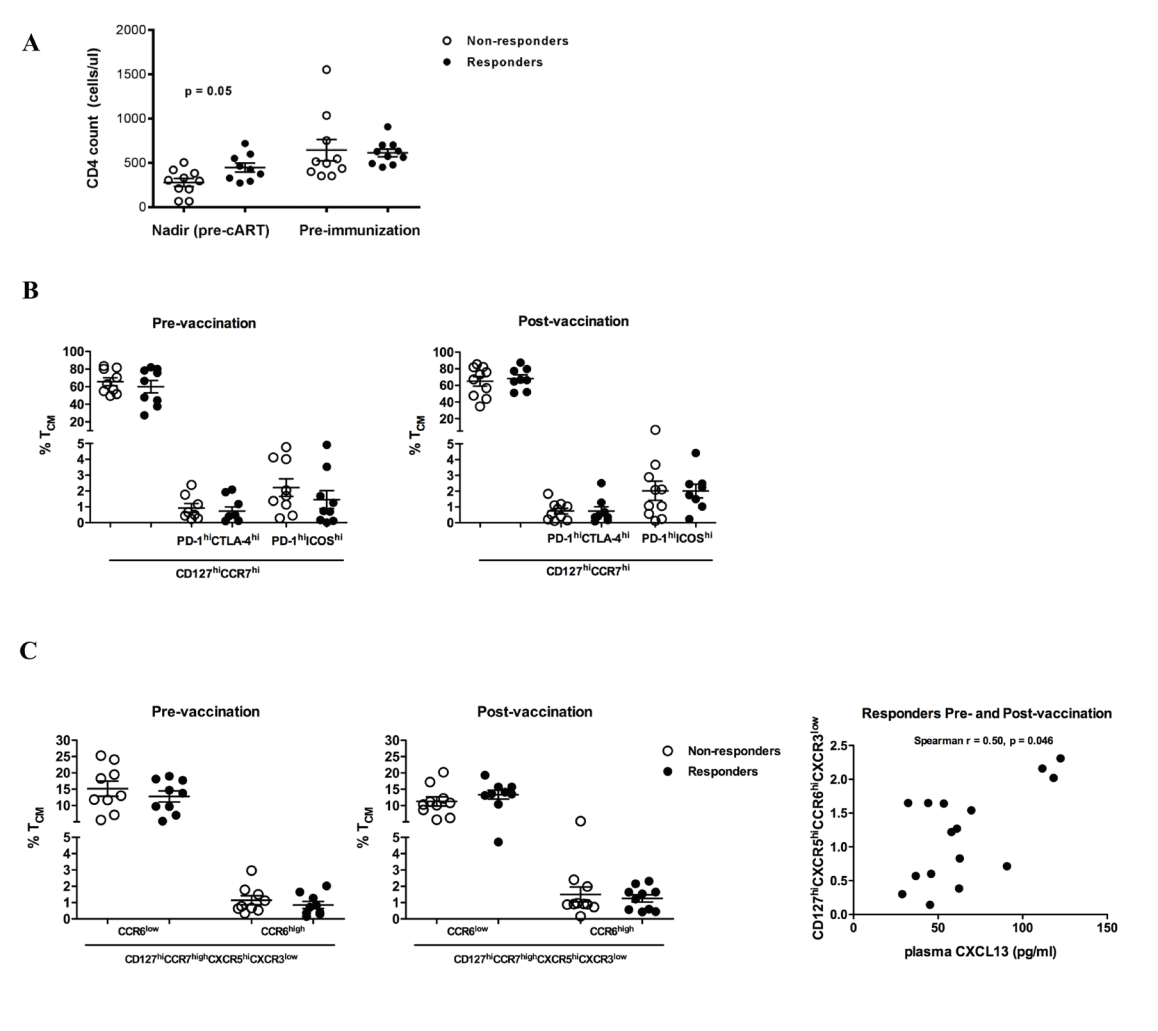
Circulating pTfh subpopulations correlate with plasma levels of CXCL-13 in HBV vaccine responders. (A) Absolute counts of CD4 T cells in non-responders and responders pre-vaccination are shown, (B) pooled data showing the relative frequencies of “activated” central memory CD127^high^CCR7^high^ CD4 T-cell subpopulations in non-responders and responders pre- and post-vaccination, (C) Pooled data showing the relative frequencies of central memory CD27^high^CD45RA^high^CD127^high^CCR7^high^ CD4 T-cell populations with respect to expression of CCR6 (*left panel*) and the correlation between CCR6^high^ pTfh cells and plasma levels of CXCL-13 is shown for vaccine responders (*right panel*). Scatterplots with mean and SEM denoted with statistical analysis performed using Mann-Whitney U test. Correlations with *P*-values determined using Spearman's rank correlation.

**Supplementary Figure 3.**
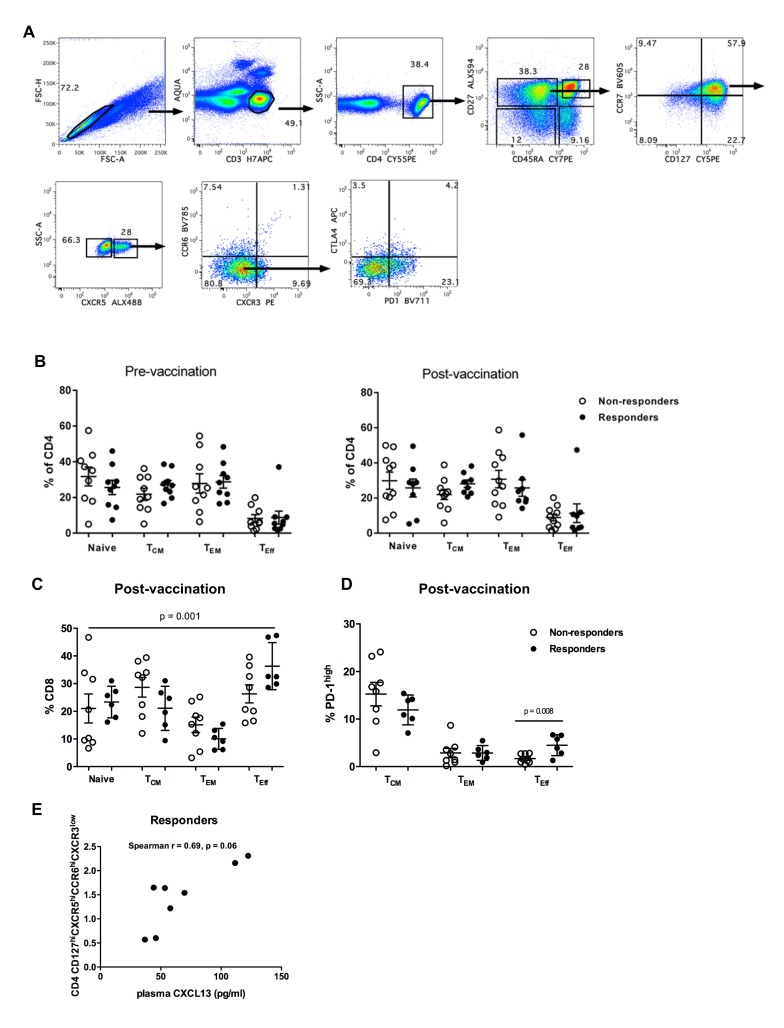
(A) Gating scheme for phenotypic analysis of CD4 and CD8 populations, (B) Pooled data showing the relative frequencies of CD4 memory subsets, judged by the expression of CD27 and CD45RA, (C) Pooled data showing the relative frequencies of CD8 memory subsets, judged by the expression of CD27 and CD45RA, (D) the frequency of PD-1^high^ CD8 T cells in memory compartments post-vaccination is shown, (E) Correlation between CCR6^high^ pTfh cells and plasma levels of CXCL-13 in responders post-vaccination. Data are comparison scatterplots with mean and SEM. P-values calculated using the Mann-Whitney U test (C) or Spearman rank correlation test (E).

Next the relative frequencies of circulating CD4 T cell subsets with potential B-cell help function [19] was analyzed. Although absolute CD4 counts didn't differ between non-responders and responders pre-vaccination, a significantly lower nadir CD4 count (pre-cART) was found in non-responders compared to responders (Figure 3A). The frequencies of memory populations defined by flow cytometry staining for CD27 and CD45RA (Supplementary Figure 3A) were similar between responders and non-responders pre- and post-vaccination for CD4 (Supplementary Figure 3B). However, post-vaccination CD8 memory T cells showed a skewed phenotype (a more differentiated, maturation profile of “memory effector-T_Eff_”) in responders (*P* = 0.001, Kruskal-Wallis test) (Supplementary Figure 3C). CD4 central memory population subsets (T_CM_, CD27^high^CD45RA^low^), to include phenotypes with an “activation” profile (PD-1^high^ICOS^high^ or PD-1^high^CTLA-4^high^), were not associated with vaccine response (Figure 3B). Next, the profiles of circulating CD4 T cell follicular helper-like cells (CD127^high^CCR7^high^CXCR5^high^CCR6^high/low^CXCR3^low^, pTfh) [19, 29] were analyzed. Contrary to responders, a trend for reduced frequency of CCR6^low^ pTfh cells after vaccination was observed in non-responders (*P* = 0.07, Wilcoxon matched pairs test) (Figure 3C, *left panel*). Although similar frequencies of CCR6^high^ pTfh cells were found between non-responders and responders (Figure 3C, *left panel*) a significant correlation was found between plasma CXCL-13 levels and the frequency of CCR6^high^ pTfh cells only in responders (Spearman r = 0.50, *P* = 0.046) (Figure 3C, *right panel* and Supplementary Figure 3E).

### Preserved Th1 adaptive responses in HBV vaccine responders

**Figure 4.**
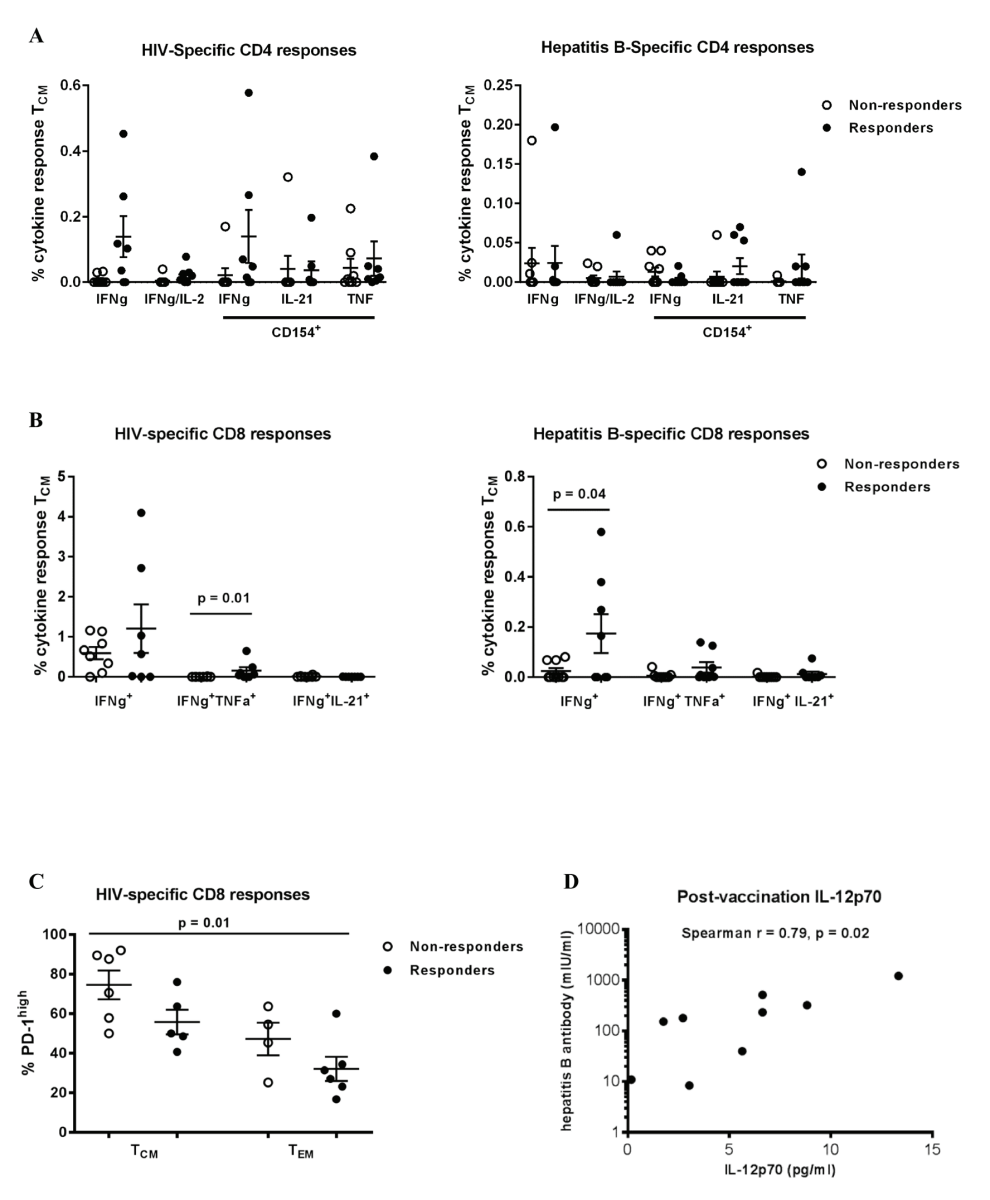
Preserved Th1 adaptive immunity in HBV vaccine responders. (A) Pooled data showing the relative frequencies of antigen-specific CD4 T cell responses measured by intracellular cytokine staining (ICS) after short *ex vivo* stimulation with HIV Gag (*left panel*) or hepatitis B surface antigen peptides (*right panel*), (B) Pooled data showing the relative frequencies of antigen-specific CD8 T cell responses measured with ICS, (C) expression of PD-1 in HIV-specific CD8 T cells in non-responders and responders (*P* = 0.001, Kruskal-Wallis test), (D) Correlation between plasma IL-12p70 levels and HbSAb titer. Data presented are scatterplots with mean and SEM with *P*-values calculated using the Mann-Whitney U test (B) or Spearman correlation test (D).

**Supplementary Figure 4.**
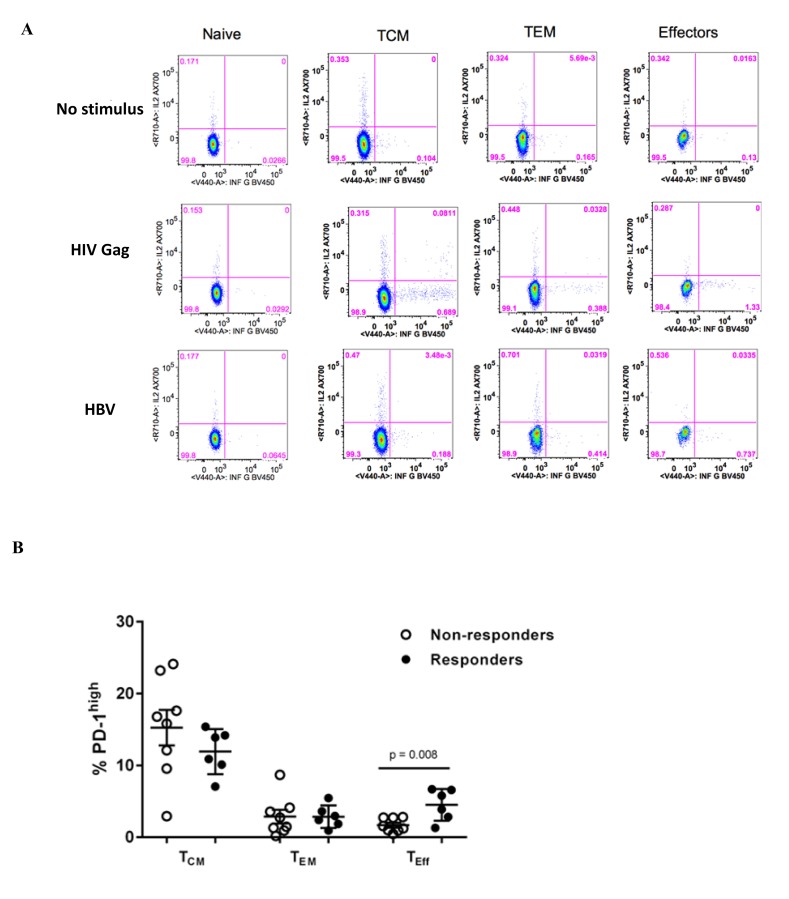
(A) Pooled data showing the relative frequencies of pTfh cells with respect to expression of CCR6 and PD-1/ICOS pre- and post-vaccination, (B) Gating scheme for analysis of CD4 and CD8 T-cell function by intracellular cytokine staining using (ICS) after stimulation with pooled HIV Gag or hepatitis B surface antigen peptides, (C) expression of PD-1 in memory CD8 T-cell subsets in non-responders and responders post-vaccination (scatterplot with mean and SEM, Mann-Whitney U test).

**Supplementary Figure 5.**
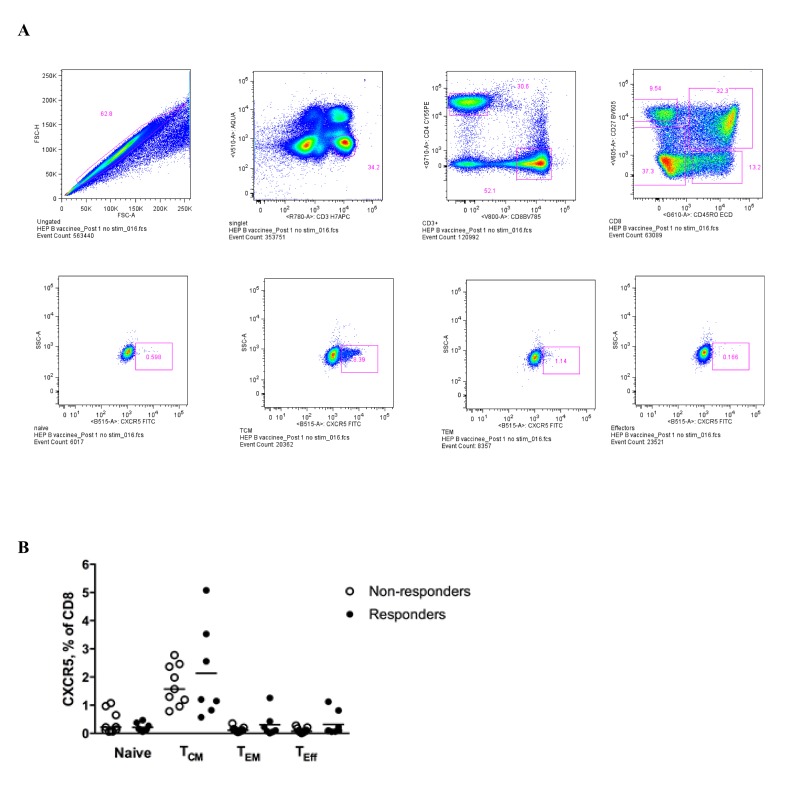
(A) Gating scheme for phenotypic analysis of CXCR5 on CD8 populations, (B) Pooled data showing the relative frequencies of CXCR5^high^CD8 T-cell populations in non-responders and responders.

We assessed T-cell function by measuring cytokine production after short (6 hours) *ex vivo* stimulation with HIV Gag and hepatitis B surface antigen pooled peptides (Supplementary Figure 4A). HIV-specific, but not hepatitis B-specific, CD4 T cells were observed more frequently after stimulation with HIV Gag or hepatitis B surface antigen peptides (Figure 4A). Although not statistically significant (*P* = 0.09), we observed an increased frequency of HIV-specific CD4 T cells among vaccine responders compared to non-responders (Figure 4A). These responses were predominantly CD27^high^CD45RO^high^ (data not shown). Both hepatitis B- and HIV-specific CD8 T cell responses were observed more frequently among HBV responders (Figure 4B). Virus-specific responses were observed more frequently in the T central memory compartment (T_CM_, CD27^high^CD45RO^high^) compared to more differentiated T cells (CD27^low^, data not shown). However, the frequencies of these virus-specific responses were not associated with the magnitude of the vaccine response (data not shown). PD-1 expression was higher on bulk, less differentiated memory CD8 T cells (T_CM_ cells, CD27^high^CD45RA^low^) while significantly higher PD-1 expression was observed on “effector memory” CD8 T cells (CD27^low^CD45RA^low^) among vaccine responders (*P* = 0.008, Mann-Whitney test) (Supplementary Figure 4B). A trend for higher PD-1 expression was observed for HIV-specific responses among vaccine non-responders, but was not statistically significant for comparison within populations (eg, T_CM_ for responders compared to non-responders), but was statistically significant across populations (*P* = 0.01, Kruskal-Wallis test) (Figure 4C). Although plasma IL-12p70, a major Th1 cytokine [30], was not associated with vaccine response, it was associated with the titer magnitude when measured post-vaccination (Spearman r = 0.79, *P* = 0.02) (Figure 4D). In light of recent observations of follicular CD8 T cell dynamics in chronic viral infection [31–33], we examined CXCR5 expression on CD8 T cell memory subsets and observed increased expression on T_CM_ compared to other subsets, but there was no difference between responders and non-responders (Supplementary Figure 5A and B). Our data point to a better-preserved adaptive antigen-specific Th1 cell immunity in vaccine responders compared to non-responders.

### Discussion

We set out to explore reasons why HIV-infected patients, with preserved or reconstituted CD4 counts > 350 cells/μl on treatment with suppressed or low viral load fail to respond adequately to standard dose and schedule hepatitis B vaccine (three doses on a 0-, 1-, and 6-month schedule of either 20 μg Engerix-B^®^ or 10 μg Recombivax-HB^®^) or maintain durable responses [5, 34, 35]. This phenomenon is observed frequently in the clinic, common in HIV infection and hemodialysis patients, and has resulted in clinical trials of alternative immunization regimens or with higher antigen dose and number of doses administered [11, 36, 37]. We observed that an immune profile prior to and/or after vaccination that is consistent with better Th1 responses was found more commonly in hepatitis B vaccine responders.

Similar general immune activation judged by sCD14 levels was found between non-responders and responders. Despite the similar immune activation, significantly higher levels of CXCL-13, a biomarker of germinal center reactions [26], were found in vaccine non-responders before vaccination, indicating a selective hyperactivity of germinal center immune reactivity in the non-responders. The similar levels of several circulating cytokines tested further support this selective profile. Furthermore, the levels of CXCL-13 were a predictor of the vaccine-induced antibody titers. Therefore, pre-vaccination vigorous germinal center reactions may negatively impact the development of HBV vaccine-induced specific humoral responses. Investigation of the relative impact of residual actively transcribed virus in lymph node areas compared to local inflammatory signals on this profile could lead to novel ways for improvement of HBV vaccine efficiency in cART-treated individuals. Whether this profile is specific for HBV vaccination or applies to other immunogens/antigens is not known and needs further investigation.

Interestingly, HBV vaccine significantly induced the circulating memory B cells expressing an IgG+ phenotype positively associated with the vaccine-induced antibody titers. Among all B-cell populations tested, this was the only population significantly affected, indicating that the HBV vaccine induced specific B cell populations in responders. No correlation between circulating CXCL-13 and vaccine-induced titers post-vaccination was found, possibly due to the time point plasmas were collected post-vaccination (7 days post-vaccination would be an appropriate time point for this purpose). However, a significant correlation was found between CXCL-13 and circulating CD4 T cell populations with increased capacity for B-cell help [19, 29] in vaccine-responders, further supporting the use of plasma CXCL-13 levels as a biomarker for germinal reactions [26]. Therefore, HBV vaccine changes the dynamics of relevant populations related to GC reactions in the responders group. Further studies, utilizing larger cohorts of relevant individuals, will establish whether such populations can be used as “biomarkers” for HBV vaccine responsiveness.

An overall better preserved antigen-specific adaptive Th1 cell immunity was found in responders compared to non-responders, especially in the antigen-specific CD8 T cell compartment for both HIV and vaccine antigens. This profile was also associated with fewer bulk and virus-specific CD8 T cells expressing an “exhausted” phenotype. The association between better preserved Th1 responses and vaccine responsiveness was further supported by the significant correlation between HBV vaccine–induced antibody titers and circulating levels of IL-12p70, a prototype member of Th1 cytokines [30]. These differences in cellular immune response may reflect earlier treatment, a higher CD4 nadir prior to treatment, or shorter duration of infection, though the two groups compared for these analyses were similar in this regard (Supplementary Table 1) [38–40]. More recently, follicular CXCR5^high^ CD8 T cells have been implicated in the control of chronic viral infection in lymphatic tissue [31, 33], to include HIV infection [32]. Differences in CXCR5 expression on peripheral T cells have been observed in non-infectious causes of altered T cell dynamics (autoimmunity and primary immunodeficiency) [41, 42]. Furthermore, recent studies have shown an increased frequency of CXCR5^high^ CD8 T cells in the lymph node follicular areas in chronic HIV [32] and SIV [43] infection. We evaluated CXCR5 expression on peripheral CD8 T cells and observed similarly increased expression in the “central memory” CD8 T cells in responders and non-responders. Further investigation is needed to confirm whether i) the better preserved circulating virus-specific CD8 T cell responses found among responders also reflect more “efficient” HIV-specific CD8 T cell immunity in the lymph nodes and follicular areas, a “sanctuary area” for HIV [31, 33], and ii) the frequency of circulating CXCR5^high^ CD8 T cells could be used as a surrogate for the accumulation of CXCR5^high^ CD8 T cells in the follicles.

In this study we did not investigate the duration of the antibody response, although one of our HBV responders had an HbSAb concentration of less than 10 mIU/mL approximately a year after completion of vaccination. This is an area of further investigation, although it is unclear whether maintenance of an adequate HbSAb concentration, once achieved, is associated with breakthrough HIV infection in treated individuals [5, 35]. Furthermore, the small sample size, given the characteristics of the subjects identified for study and the availability of archived specimens for testing, did not allow for further analysis of T-cell populations that could have an impact on the development of B-cell responses. However, our study points to possible mechanisms for the responsiveness to HBV vaccination in HIV infected individuals [4, 5] even after cART treatment [4, 6] and provides a rational base for future studies focusing on the germinal center dynamics that could direct HBV vaccine efficacy.

## SUPPLEMENTARY MATERIALS

**Supplementary Table.** Characteristics of vaccine cohort subjects

Subject	HIV positive date	Age (at vaccination)	Sex	CD4 nadir (before cART)	HbSAb Negative Date	Pre-Immunization Specimen Date	CD4 count	Viral Load (copies/ml)	Third Vaccination Date	HbSAb Positive Date	Post-Immunization Specimen Date
**HBV Non-responders (n=10)**											
221192	5/1/2001	30	Male	304	6/11/2001	6/18/2001	401	<400	3/15/2002	6/10/2002	6/10/2002
269017	8/14/2003	24	Male	210	10/27/2003	11/3/2003	1554	440	5/15/2004	11/29/2004	11/29/2004
341027	9/19/2002	24	Male	295	10/28/2002	11/4/2002	352	<50	10/15/2003	6/14/2004	12/15/2003
371003	10/22/2003	34	Male	330	12/8/2003	12/15/2003	514	831	8/15/2004	1/10/2005	1/10/2005
478307	9/30/2002	23	Male	203	10/21/2002	10/28/2002	545	310	11/15/2003	2/24/2004	2/23/2004
538900	2/5/2002	37	Male	383	4/1/2002	4/8/2002	435	<400	4/15/2003	12/8/2003	12/8/2003
702187	1/27/1992	44	Male	65	6/5/2006	6/5/2006	752	<50	1/15/2007	6/4/2007	6/4/2007
786612	1/31/1997	67	Male	504	7/19/2006	7/17/2006	1036	<50	8/15/2007	9/16/2008	3/3/2008
825051	3/9/1987	32	Male	420	9/23/1996	11/6/1996	494	891	4/15/2000	4/16/2001	4/16/2001
915537	3/22/1999	54	Male	66	4/3/2000	4/3/2000	352	58	10/15/2000	4/30/2001	4/30/2001
**HBV Responders (n=10)**											
157182	11/12/1996	40	Female	543	2/1/1999	2/1/1999	573	<400	1/15/2000	4/24/2000	4/24/2000
324067	6/30/1989	42	Male	327	5/20/1996	2/11/2002	632	<50	12/15/2003	9/13/2004	3/1/2004
403888	2/17/2009	41	Female	599	4/6/2009	4/13/2009	907	371	10/15/2009	3/22/2010	3/22/2010
474859	2/11/2002	27	Male	374	3/11/2002	2/24/2003	493	527	10/15/2003	3/29/2004	3/29/2004
672156	10/26/1994	36	Male	462	3/12/2002	6/10/2002	628	792	2/15/2003	6/23/2003	6/23/2003
673743	2/5/1999	40	Male	551	2/23/1999	10/10/2000	702	<50	1/15/2010	3/8/2012	8/9/2010
832387	4/24/1995	31	Female	273	7/8/1998	7/8/1998	562	<400	10/15/1998	12/16/1998	12/16/1998
882477	10/13/1999	34	Male	292	11/15/1999	10/26/2000	450	106	8/15/2002	1/22/2007	5/13/2003
942173	8/12/1997	34	Male	719	3/8/1999	3/8/1999	700	<400	3/15/2000	4/11/2000	3/12/2001
965852	3/22/2005	27	Male	426	6/19/2006	12/11/2006	478	<50	7/15/2007	6/30/2008	12/17/2007

Abbreviations: cART, combination antiretroviral therapy; HBV, hepatitis B vaccine; HbSAb, hepatitis B surface antibody
